# Asymmetrical Effects of Language Mixing in Bilingual Children’s Word Learning

**DOI:** 10.3390/bs16091547

**Published:** 2026-09-01

**Authors:** Minjeong Kang, Youngon Choi

**Affiliations:** Department of Psychology, Chung-Ang University, 84 Heukseok-ro, Dongjak-gu, Seoul 06974, Republic of Korea; wonjung20@cau.ac.kr

**Keywords:** bilingualism, code-switching, word learning, language distance, language dominance

## Abstract

Language mixing is a common feature of bilingual environments, yet its effects on bilingual children’s word learning remain inconsistent across studies. The present study examined how language mixing affects novel word learning in 3- to 4-year-old Korean–English bilinguals, extending previous work to an understudied language pair with a greater language distance than those studied previously. Following an established experimental paradigm, children were taught novel words embedded in either single-language or mixed-language sentences during a looking-while-listening task. For half the children, carrier sentences introduced the novel words in their dominant language; for the other half, they were introduced in their non-dominant language. Overall, children successfully mapped the novel word to referents in the single-language context but showed difficulty doing so in the intra-sentence code-switched language context. However, learning patterns diverged by the dominance of the carrier language; when carrier phrases were presented in a non-dominant language, language mixing significantly affected children’s novel word learning, supporting learning only in a single-language context. No difference in language mixing was observed when the carrier language was dominant. These findings partially replicate and extend prior work on the effects of language mixing on young bilingual learners’ word learning, suggesting that the costs of language mixing vary across learning contexts, depending on the specific switching directionality between two distant languages.

## 1. Introduction

Language mixing, also referred to as code-switching, is a natural and widespread feature of communication in multilingual communities (Myers-Scotton, 2017). It occurs not only in conversations among adults, but also in speech directed to children: parents have been shown to mix languages when interacting with their children, and code-switching serves functions such as attracting children’s attention or supporting vocabulary learning in some contexts (Bail et al., 2015; Kremin et al., 2022; Ruan et al., 2023). With bilingualism becoming increasingly common, a growing number of children are raised in language-mixed learning environments, with two languages switched within or across utterances (Bail et al., 2015; Byers-Heinlein, 2013; Kremin et al., 2022). How this mixed-language input affects children’s word learning is one of the important questions in the study of bilingual development, as children’s word learning is heavily shaped by the quantity and quality of language input (Goodman et al., 2008; Hoff et al., 2012; Hurtado et al., 2008; Weisleder & Fernald, 2013).

Real-time language processing, such as word recognition, has been shown to be negatively influenced by code-switching, whereby bilingual children are slower and less accurate in identifying target referents in intra-sentential mixed-language sentences than in single-language sentences (Byers-Heinlein et al., 2017; Morini & Newman, 2019; Potter et al., 2019). Pupil dilation as a physiological index of cognitive load was shown to increase as a result of language switches within sentences, suggesting that intra-sentential language mixing engages additional cognitive resources when young learners process and recognize words within the mixed-language context (Byers-Heinlein et al., 2017).

Morini and Newman (2019) also showed that while bilingual toddlers’ word recognition typically benefits from hearing words in sentence frames rather than in isolation, this sentence-context advantage was present only in single-language sentences. In mixed-language sentences, recognition accuracy was not better than when words were presented alone, suggesting that language mixing eliminated or reduced the advantage provided by sentential context. Processing costs associated with language mixing have also been documented in school-age children, who show slower processing speeds when listening to mixed-language sentences relative to single-language sentences, with these costs linked to individual differences in cognitive control (Gross et al., 2019).

According to the Bilingual Interactive Activation models (A. Dijkstra & van Heuven, 1998; T. Dijkstra & van Heuven, 2002), bilinguals store both languages within a single integrated lexicon, and both languages are simultaneously co-activated during comprehension regardless of which language is used. When language switching occurs, the system modulates these competing activation levels—suppressing irrelevant language representations while maintaining target language processing—and this additional regulatory effort gives rise to processing costs. The Inhibitory Control Model (Green, 1998) similarly proposed that language switching requires active suppression of the non-target language to prevent interference, a process that demands additional cognitive resources.

Interestingly, the mixed-language processing costs were found to be asymmetrical in bilingual learners: difficulties arose primarily when the switch occurred from the dominant language into a non-dominant language, manifested as slower convergence on the target referent (Potter et al., 2019) and greater pupil dilation (Byers-Heinlein et al., 2017) during dominant-to-non-dominant switch conditions compared with the opposite condition. Similar asymmetrical processing costs were also reported in unbalanced bilingual adults’ sentence comprehension (Bultena et al., 2015a, 2015b).

If these processing costs extend to word learning contexts, language mixing may hinder children’s acquisition of novel words. Several studies have examined the effect of language mixing on word learning but yielded mixed results. Byers-Heinlein et al. (2022) taught two groups of 3- to 4-year-old bilingual children novel words presented in either a single-language or mixed-language context. Specifically, code-switching occurred within the sentence for a familiar word for the child before the novel word was introduced (e.g., There is a *dog/chien* on the **teelo**.). French–English bilingual children successfully learned novel words from single-language sentences but failed to do so when the words appeared after a code-switched familiar word in mixed-language sentences. Spanish–English bilingual children, by contrast, showed no reliable word learning in either condition, showing variable consequences across different bilingual populations. Whether asymmetric learning cost related to dominant-to-non-dominant switches also influences word learning beyond word recognition was not directly tested in this study.

Furthermore, in other learning situations with slightly older children, mixed-language input did not necessarily hinder learning (Libersky et al., 2025) and even appeared beneficial for learning (e.g., Kaushanskaya et al., 2023) among 4- to 5-year-old Spanish–English bilingual children. These inconsistent findings suggest that the effects of language mixing on word learning cannot be characterized as straightforwardly negative or positive, but are likely modulated by a range of factors, including task type and demand, the specific language pair involved, and the broader sociolinguistic context in which learning takes place.

These mixed findings may also reflect the limited diversity of the bilingual samples examined in prior work. Because these effects appear to depend on multiple factors, examining a broader range of bilingual population is important for clarifying when and how language mixing affects word learning. Niu et al. (2024) drew attention to one such dimension, language distance. Language distance refers to the degree of difference between two languages, encompassing dimensions such as genealogy, typology, grammar, and semantics (Chiswick & Miller, 2005; Cysouw, 2013). Niu et al. (2024) examined the effects of language mixing on novel word learning in 4-year-old Mandarin–English and Mandarin–Japanese bilingual children, using the same experimental paradigm as in Byers-Heinlein et al. (2022). The negative effect of language mixing was generally replicated: both groups showed poorer word learning in the mixed-language condition relative to the single-language condition. Additionally, the Mandarin–English group demonstrated significantly better word learning outcomes than the Mandarin–Japanese group, whose two languages are typologically closer than Chinese and English. The authors attributed this pattern to heightened cross-language competition: when two languages share more structural and phonological overlap, their representations compete more strongly, placing greater demands on processing mixed-language input. These results suggest that the impact of language mixing on word learning may vary as a function of the distance between the two languages, highlighting the importance of considering language distance as one of several factors that may contribute to variability in bilingual word learning.

However, the findings on language mixing and word learning in bilingual children have largely come from French–English and Spanish–English bilingual populations, both of which belong to the Indo-European language family. The study by Niu et al. (2024) was the only exception that examined the effects of code-switched input in Chinese bilingual populations. However, these Chinese 4-year-olds acquired the two languages within the preceding year in a preschool setting, requiring the effect of language distance (see also Gallo et al., 2024; Ljungberg et al., 2020) to be tested and confirmed with other populations, such as simultaneous bilingual learners.

In the present study, we aimed to investigate the effects of language mixing on word learning and its potential modulation by language distance by testing an untested bilingual population with two languages that are distant relative to the majority language pairs studied thus far. Specifically, we targeted 3- to 4-year-old Korean–English bilingual children. Korean differs substantially from English in terms of genealogy, grammar, and phonology (K. Kim, 2016; M. Lee, 2015; J. Lee & Min, 2015; Park & Yoon, 2021; Yeo et al., 2023) and is as distant from English as Chinese and Japanese, as shown across various distance indices (Chiswick & Miller, 2005; Cysouw, 2013). Using a quantitative index of language distance that takes into account learnability and genealogical classification, Chiswick and Miller (2005) found that Korean is considerably more distant from English than French or Spanish (Korean: 1.0; French: 2.50; Spanish: 2.25; lower scores indicate greater distance from English, with the range between 1 and 3, with 1 being harder to learn and 3 being easier to learn by English native speakers), and that Korean has no genealogically close relative among the world’s major languages. Consistent with this characterization, Cysouw (2013) confirmed that Korean is markedly more distant from English than Spanish or French across multiple dimensions: in particular, Korean showed much lower typological similarity to English (Korean: 0.453; Spanish: 0.615; French: 0.662). Therefore, testing Korean–English bilingual learners can shed further light on the role that code-switched input plays in learning as well as how language distance may modulate this effect.

Importantly, to allow for comparisons with the French–English and Spanish–English populations tested in Byers-Heinlein et al. (2022), we closely replicated their design and directly borrowed their visual stimuli, teaching our bilingual children novel words in similar single-language and mixed-language contexts, as the child’s word learning is measured using the same looking-while-listening paradigm. Across participants, carrier phrases of novel words were presented in either the child’s dominant or non-dominant language, as in Byers-Heinlein et al. (2022). Based on the language distance hypothesis (Niu et al., 2024; see also Gallo et al., 2024; Ljungberg et al., 2020), Korean–English bilingual children, whose two languages are considerably more distant than the French–English and Spanish–English pairs examined in prior work, would be expected to undergo relatively less interference from language mixing and thus learn novel words more successfully in the mixed-language condition.

Additionally, given prior evidence that language mixing imposes asymmetrical processing costs depending on bilinguals’ language dominance in word recognition tasks (Byers-Heinlein et al., 2017; Potter et al., 2019), we aimed to explore whether a similar dominance-related asymmetry would exist in the context of novel word learning by comparing learning patterns based on language dominance.

## 2. Materials and Methods

We modeled the design and materials closely after those used in Byers-Heinlein et al. (2022) to test the effect of language mixing under the same conditions as in that study. The visual stimuli were adapted from Byers-Heinlein et al. (2022), and the sentences were constructed to reflect the visual stimuli and experimental setting the study.

### 2.1. Participants

The final sample included 24 Korean–English bilingual learners aged 3 to 4 years (14 girls; M = 42.02 months, range = 36–50) who received regular exposure to both Korean and English from early in life. According to an a priori power analysis using G*Power (Version 3.1.9.6, Faul et al., 2007, 2009), with 80% power and α = 0.05 (two-tailed), 19 participants were required to detect the reported effect size (*d*_z_η = 0.69; Byers-Heinlein et al., 2022) in a paired-samples *t*-test. However, we tested additional children who were scheduled to participate. All children (*M*_age_ = 41.88 months, *SD* = 4.22, boys = 10) resided in Seoul and its metropolitan area and had acquired Korean and English from birth, except 6 children who began learning English later than Korean (sample mean age of English acquisition = 3.58 months, range = 0–20 months). Data from children who scored below the 10th percentile on the vocabulary test were not included for final analyses.

Of the final sample, 17 children were identified as Korean-dominant and 7 as English-dominant, based on the caregiver reports on the adapted version of the Language and Social Background Questionnaire (Anderson et al., 2017). Mothers’ years of education averaged 17.29 years (*SD* = 2.26), indicating an overall high level of parental education. Demographics, the child’s vocabulary scores, and language mixing scores are summarized in Table 1.

Participants were recruited through a database of families who agreed to participate in our research, word of mouth, online communities, and social media. The participating families visited our lab in Seoul, South Korea, and data were collected from 2025 to 2026. The experiment was approved by the Chung-Ang University Institutional Review Board (1041078-20240826-HR-231). Written consent was obtained from parents after they received an explanation of the study, and their child verbally agreed to participate.

### 2.2. Materials

We followed the same design and borrowed visual stimuli from Byers-Heinlein et al. (2022) but created new auditory stimuli and novel words. We created two novel words, *Jonu* and *Nombi*, based on the nativeness ratings of English and Korean native speakers (15 each). Specifically, the adult raters were asked to rate how much each word sounded like English or Korean on a 5-point scale from 1 = not at all likely to 5 = very likely. The final two words were chosen from the list of words that these adult listeners perceived as equally likely to be Korean or English, taking into account the phonological salience of each carrier language context.

**Figure 1:**
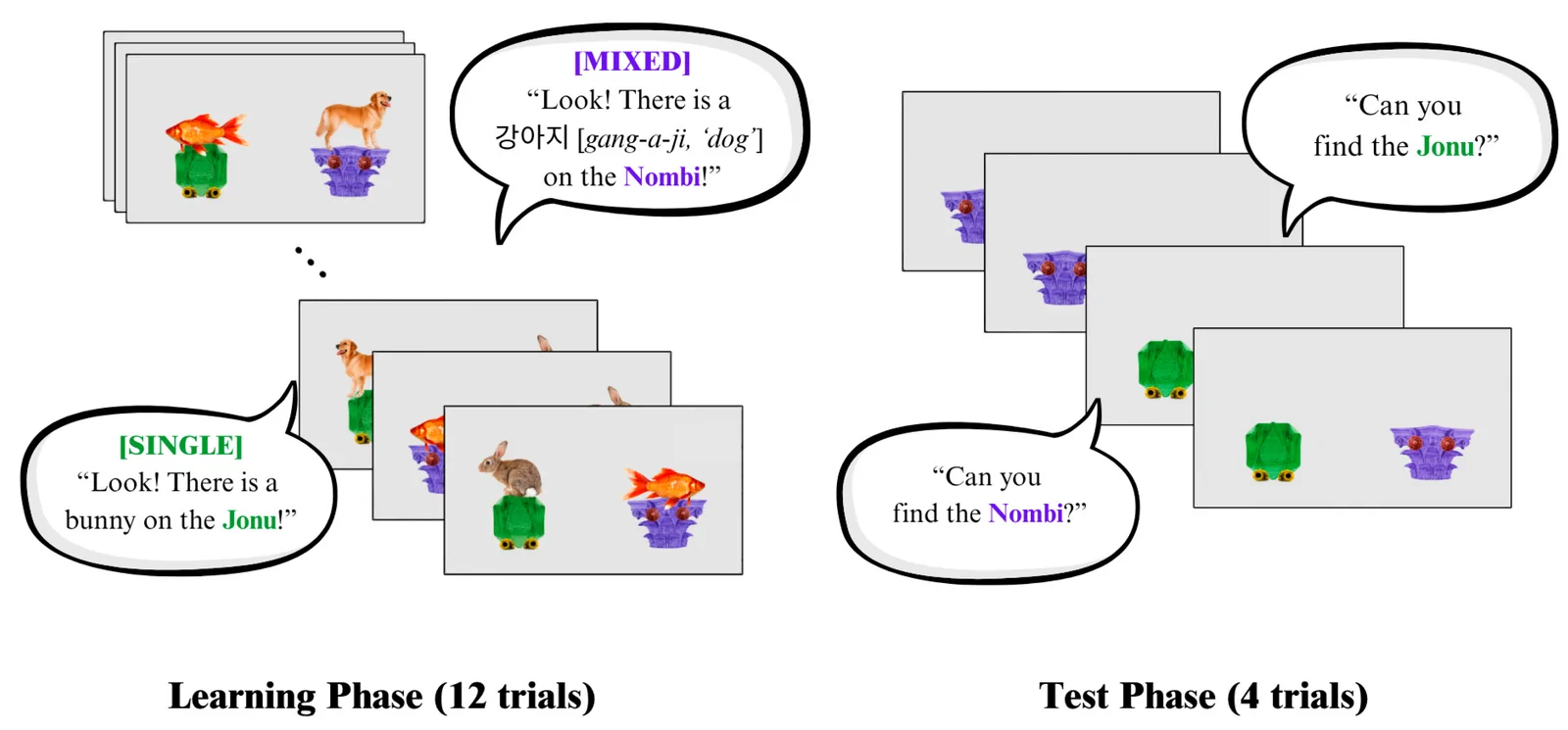
Auditory and visual stimuli for learning and test trials. The visual stimuli were reproduced from Byers-Heinlein et al. (2022), available at https://osf.io/q2nzr/ (accessed on 3 October 2024, CC BY 4.0).

During learning trials, participants saw two familiar animals (e.g., a dog and a rabbit) sitting on two novel objects (see Figure 1). The novel label was presented in a carrier sentence across 12 learning trials. For half of the learning trials, carrier sentences were composed of a single language (Korean or English) (e.g., “Look! There is a dog on the *Nombi*!” or “봐 봐! 강아지가 *놈비* 위에 있어! [*Bwa bwa! Gang-a-ji-ga Nombi wi-e iss-eo!*]”), and the remaining half contained both languages (e.g., “Look! There is a 토끼 [*tokki*] on the *Jonu*!” or “봐 봐! Bunny 가 *조누* 위에 있어! [*Bwa bwa! Bunny-ga Jonu wi-e iss-eo!]*”). The novel word always appeared after the familiar word, as in Byers-Heinlein et al. (2022). During the test trials, target words were presented only in the main carrier language sentences (e.g., “Can you find the *Nombi*?” or “어떤 게 *조누*야? [*Eo-tteon ge Jo-nu ya*?]”).

All sentences in the experiment were recorded by a female Korean–English bilingual speaker using child-directed speech. Each sentence was recorded several times, and the token that best matched the intended clarity and delivery was selected. Following Byers-Heinlein et al. (2022), the familiar words were consistently pronounced according to the phonology of their respective languages, whereas the novel target words were pronounced according to the phonology of the carrier phrase’s language. The recordings were processed and edited in Audacity (Version 3.6.4; Audacity Team, 2024) and Praat (Version 6.4.22; Boersma & Weenink, 2024).

#### Language Experience and Vocabulary Assessments

Children’s language backgrounds were assessed using an adapted version of the Language and Social Background Questionnaire (LSBQ; Anderson et al., 2017), with items modified to be applicable to young children. Mothers also completed the Language Mixing Questionnaire (Byers-Heinlein, 2013) to report on the extent of language mixing.

Children were administered two standardized language assessments: the Peabody Picture Vocabulary Test (4th edition; PPVT-IV; Dunn & Dunn, 2007) for English vocabulary and the Receptive and Expressive Vocabulary Test-Receptive (REVT-R; Y. T. Kim et al., 2009) for Korean word acquisition. Both tests asked the child to select the picture that best describes the target word. We obtained receptive vocabulary scores from both tests. Table 1 summarizes the child’s language background and vocabulary scores.

### 2.3. Procedure

Upon arrival at the laboratory, the experimenter explained the study and its procedures and obtained caregivers’ consent and the child’s verbal agreement. One of the receptive vocabulary assessments was administered before the word learning experiment, and the other after. While the children were completing these language assessments, their caregivers completed questionnaires about their children’s language background and language mixing.

The word learning task procedure was identical to that of Byers-Heinlein et al. (2022). Children sat on their caregiver’s lap about 70 cm away from a monitor (68 × 122 cm) in a sound-attenuated room. The auditory stimuli were presented at an average volume of 70 dB. To prevent the caregiver’s influence on the children’s behavior, caregivers wore sunglasses that blocked two-thirds of the screen view and headphones that played popular Korean songs. They were also asked not to interact or speak to their child during the experimental session.

Children were randomly assigned to one of four experimental orders. They completed 12 learning trials—6 single-language and 6 mixed-language trials—followed by 4 test trials. Depending on the assigned condition, the carrier sentences were presented in either Korean or English. Twelve children completed the experiment with carrier phrases in their dominant language. The other 12 completed it in their non-dominant language.

Across 12 learning trials, pairs of familiar and novel objects were presented side by side on the screen. After 2000 milliseconds (ms), children were asked to look at the familiar target located above the novel object. The auditory stimulus ended at 6000 ms. Each learning trial lasted 8000 ms. Each novel object was consistently labeled using either single-language or mixed-language sentences. The assignment of names to sentence types and the location of objects were all counterbalanced across children. Each novel object served as the target for half of the trials and as the distractor for the other half. The order of the learning trials was semi-randomized.

During the four test trials, only the two novel objects were presented on the screen. After 2000 ms, the children were instructed to find the referent object corresponding to the target label. The auditory stimulus ended at 4000 ms, and each test trial lasted 6000 ms. Each novel object appeared twice as the target and twice as the distractor. The lateral position of the objects was counterbalanced both within and between subjects.

### 2.4. Coding

Children’s eye gaze was recorded with a video camera located below the screen at a frame rate of 29.97 Hz. Eye fixation patterns were manually coded frame by frame as looking left, right, middle, or away for the entire duration, using Adobe Premiere Pro (Version 25.6; Adobe Inc., 2025). Two coders fully double-coded 33% of the videos, reaching 99.04% agreement. The data were split and coded by the two coders after establishing the coding agreement.

### 2.5. Analysis Plan

For all analyses, the dependent measure was the mean proportion of target-looking time across trials. It was calculated as the total time children spent looking at the target divided by the total time they spent looking at both the target and distractor objects. Looking measures were analyzed for learning trials to track whether the child properly engaged in learning the labels of the target referent, and for test trials to examine the outcome of word learning.

During the learning trials, the target region included both the familiar animal and the novel object (e.g., a bunny atop a novel item) because looking at these two items was difficult to code separately, given their close proximity. During test trials, the target region included only the target referent, the novel object. The analysis window for both learning and test trials was set from 360 ms after the onset of the target word (a familiar word for learning trials and a novel label for test trials; e.g., bunny, Jonu) to 2000 ms after the word ended (Swingley, 2012). Trials in which the total looking time to the screen fell below 750 ms within this window were excluded from the final analyses.

All analyses were conducted using R (version 4.5.2; R Core Team, 2025). First, we performed one-sample *t*-tests to examine whether children looked at the target region during learning trials reliably above chance (0.5), assessing whether they attended to the target as they learned the novel label. We then ran a paired-samples *t*-test to examine whether children’s word learning during the test differed by language mixing, as reported in Byers-Heinlein et al. (2022). To determine the word learning outcomes in each condition, one-sample *t*-tests were also performed to examine children’s looks at the target against chance (0.5) under each condition. Because the dependent measure (mean looking proportion) was bounded between 0 and 1, a logit transformation was applied to meet the assumption of normality for the residuals. A Shapiro–Wilk test confirmed that, while the model using raw data violated the normality assumption (W = 0.966, *p* = 0.028), the logit-transformed model satisfied it (W = 0.978, *p* = 0.179). All inferential tests were therefore conducted on logit-transformed values.

To further examine the effects of other factors on word learning, linear mixed-effects models (LMEs) were fitted using the *lme4* package (version 2.0-1; Bates et al., 2015). We used a model comparison approach to select the best fitting model. The base model (Model 1) included language mixing (single vs. mixed), the dominance of the carrier phrase language (dominant vs. non-dominant), and their interaction as fixed effects, with the mean target-looking proportion during the learning phase (attention during learning) as a covariate and a random intercept for participants. Language mixing was a within-participant factor, whereas language dominance of the carrier phrase was a between-participant factor. Both predictors were sum-coded to ensure that the main effects could be interpreted as the effect of one factor at the grand mean of the other factor and to allow for meaningful interpretation of the interaction term. The dominance of the carrier phrase language was included in this model to explore its potential effect on bilingual word learning, as language dominance results in asymmetrical processing costs in bilingual children’s word recognition (Byers-Heinlein et al., 2017; Potter et al., 2019). To test whether additional covariates improved model fit, children’s English and Korean receptive vocabulary scores were added in Model 2 and the mother’s language mixing score was added in Model 3. The models were compared using likelihood ratio tests to determine whether added factors provide a better fit. Where significant main effects or interactions were identified in the final model, pairwise comparisons were conducted using the *emmeans* package (Lenth & Piaskowski, 2026).

Finally, to explore whether individual differences in children’s word learning were related to child-level and environmental measures, we computed Pearson correlations among children’s age, vocabulary in the dominant and non-dominant languages, mother’s language mixing scores, mother’s education, and children’s attention during learning trials and target looking during the test in each condition. Because these correlational analyses were exploratory and involved a large number of comparisons, *p*-values were adjusted using Bonferroni correction.

## 3. Results

Of the 24 participants in the final sample, 1 participant’s data was excluded from the test-phase analysis of the mixed-language condition because the child’s looking times fell below 750 ms in both test trials. As a result, the single-language condition included data from all 24 participants, whereas the mixed-language condition included data from 23 participants. Due to an equipment error, one child’s data were not recorded for learning trials. Therefore, 23 children were included in the final analyses of learning trials.

As planned, we first examined whether the children were successfully attending to the target referent during learning. Two-tailed one-sample tests showed that children looked at the target reliably above chance (0.5) in both the single-language [*M* = 0.70, *SD* = 0.15, *t*(22) = 6.64, *p* < 0.001, *d* = 1.39] and the mixed-language [*M* = 0.70, *SD* = 0.13, *t*(22) = 6.97, *p* < 0.001, *d* = 1.45] trials. This suggested that children were equally likely to attend to the target referent in both single- and mixed-language learning contexts.

**Figure 2:**
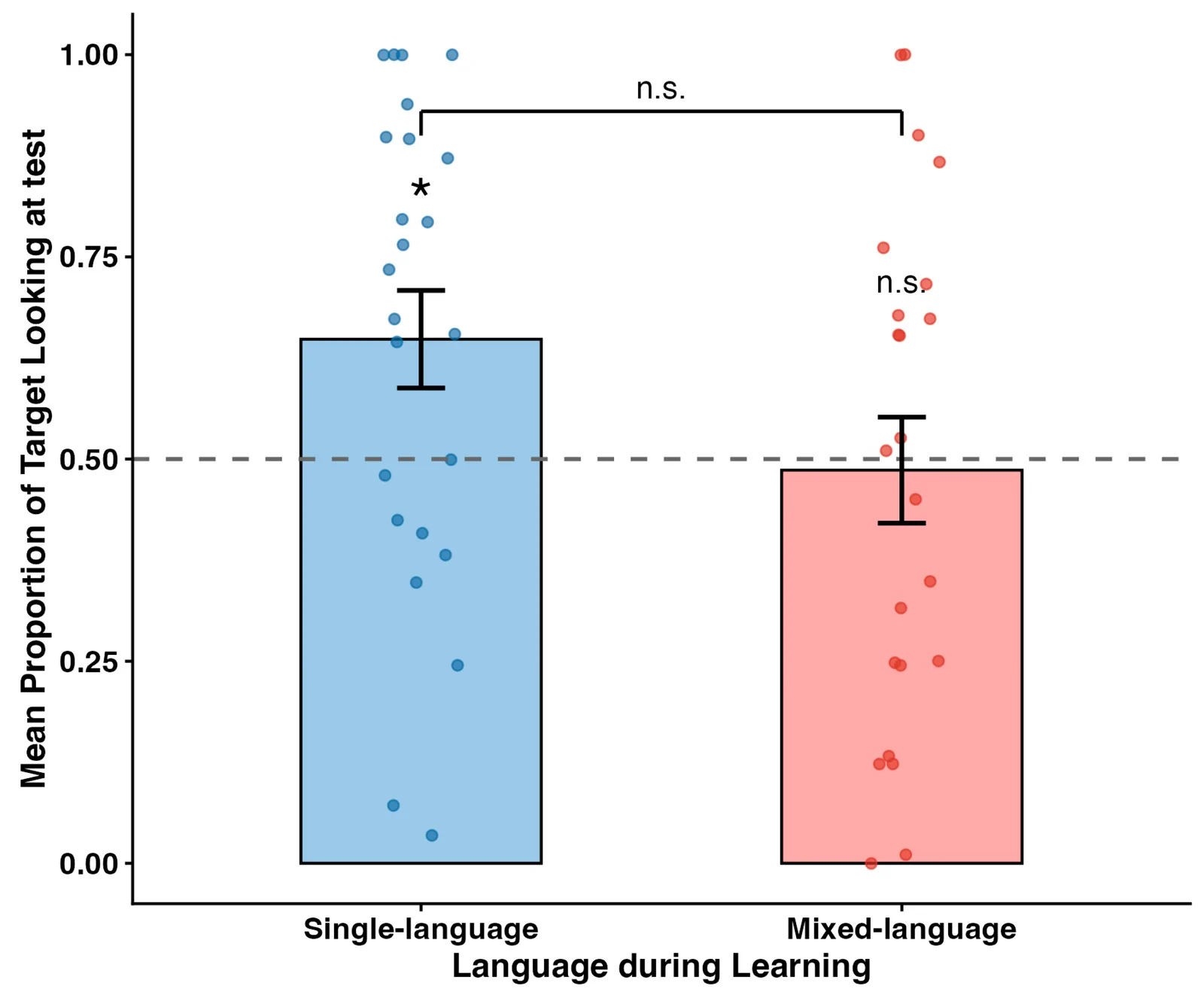
Mean proportion of time spent looking at target object during test phase, split by language mixing condition. Error bars represent standard errors of mean. * *p* < 0.05, n.s. = not significant.

A two-tailed paired-samples *t*-test was then performed to examine the effect of language mixing on the mean proportion of time spent looking at targets during the test. The effect of language mixing approached significance [*t*(22) = 1.70, *p* = 0.10, *d* = 0.36]. To test children’s looks against chance, separate one-sample *t*-tests were conducted for single- and mixed-language conditions. Children’s looking time toward the target referent was significantly above chance [*M* = 0.65, *SD* = 0.30, *t*(23) = 2.46, *p* = 0.022, *d* = 0.50] after they learned the label in the single-language context. Conversely, looking proportions did not differ significantly from chance in the mixed-language sentences [*M* = 0.49, *SD* = 0.31, *t*(22) = −0.21, *p* = 0.836, *d* = −0.044] (see Figure 2).

Finally, the linear mixed-effects analyses revealed a marginal main effect of language mixing (*β* = −1.391, *SE* = 0.823, *p* = 0.096) and a significant interaction between language mixing and the dominance of the carrier sentence language (*β* = 4.654, *SE* = 1.646, *p* = 0.006). Adding children’s receptive vocabulary scores [*χ*^2^(2) = 2.24, *p* = 0.327] and the mother’s language mixing score [*χ*^2^(1) = 2.65, *p* = 0.103] to the base model did not significantly improve model fit (see Table 2).

**Figure 3:**
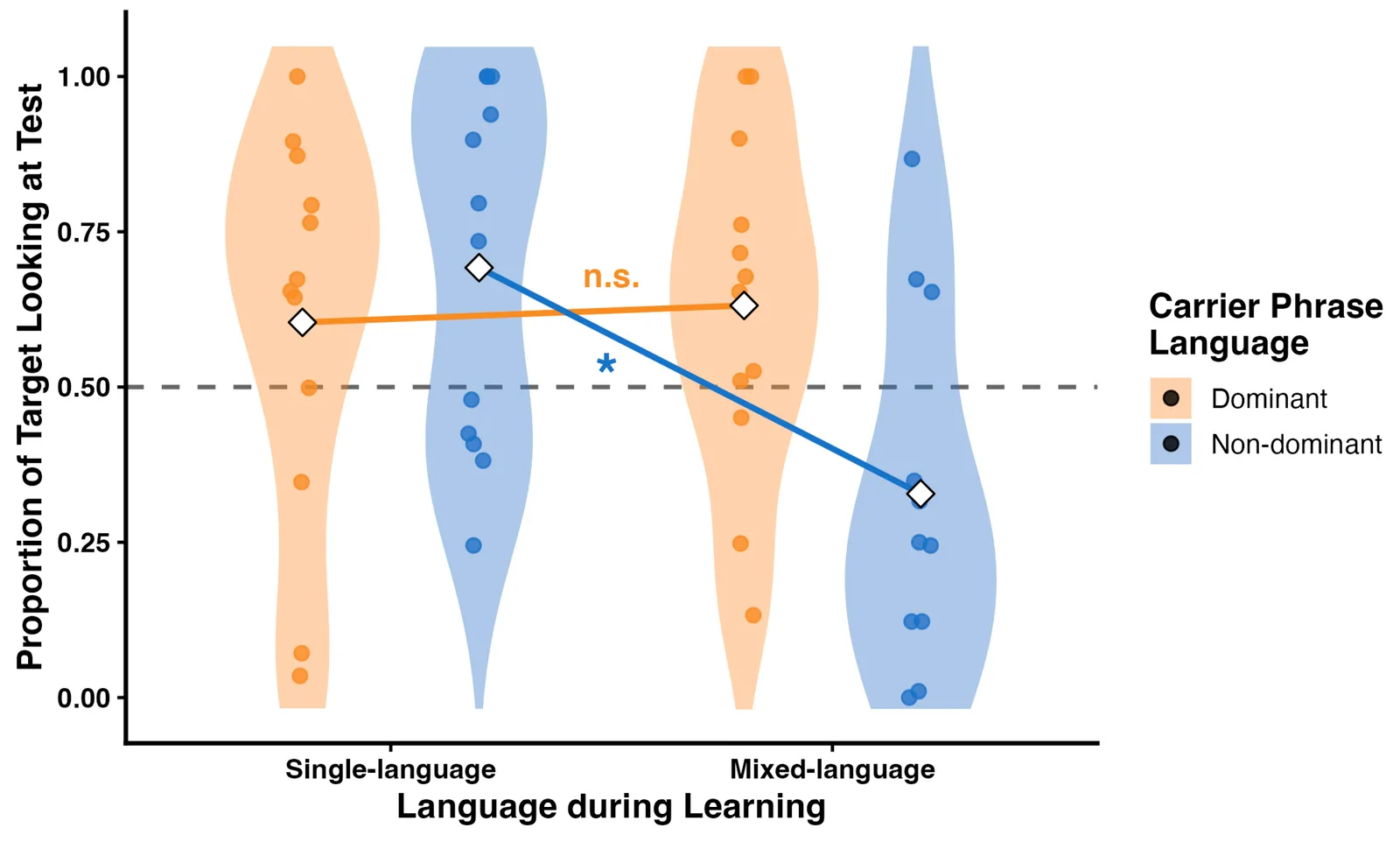
The distribution of target looking proportions during the test phase as a function of language during learning and carrier phrase language. Violin plots show the distribution of target looking proportions in each condition. Dots represent individual participants. White diamonds indicate observed condition means, and lines connect the means within each carrier phrase language group. The dashed line indicates chance level (0.5). The figure displays raw proportions, whereas all statistical analyses were conducted on logit-transformed data. Asterisks indicate a significant within-group difference between conditions based on Bonferroni-corrected post hoc comparisons from the linear mixed-effects model. * *p* < 0.05; n.s. = not significant.

We conducted pairwise comparisons using the *emmeans* package with Bonferroni correction (see Figure 3). The result showed that when the carrier phrase was presented in the child’s dominant language, test performance did not differ between the single- and mixed-language conditions [*t*(60.9) = 0.86, *p* = 1.000]. In contrast, when the carrier phrase was presented in the non-dominant language, the target looking proportion was significantly lower in the mixed-language condition compared to the single-language condition [*t*(63.0) = −3.02, *p* = 0.014]. Exploratory one-sample *t*-tests showed that time spent looking at the target did not differ significantly from chance in either the single-language [*M* = 0.60, *SD* = 0.31, *t*(11) = 1.01, *p* = 0.335] or the mixed-language condition [*M* = 0.63, *SD* = 0.27, *t*(11) = 1.70, *p* = 0.117] when the carrier language was dominant. By comparison, when the carrier language was not the dominant language, time spent looking at the target was significantly above chance in the single-language condition [*M* = 0.69, *SD* = 0.29, *t*(11) = 2.39, *p* = 0.036], whereas it showed a tendency toward below-chance performance in the mixed-language condition [*M* = 0.33, *SD* = 0.29, *t*(10) = −1.92, *p* = 0.084].

Further tests confirmed that there were no significant differences in age [*t*(21.87) = −0.43, *p* = 0.674], receptive vocabulary in Korean [REVT; *t*(21.23) = −0.80, *p* = 0.431] or in English [PPVT-4; *t*(18.31) = 0.12, *p* = 0.904], mother’s language mixing scores [*t*(21.95) = 0.46, *p* = 0.648], or mother’s years of education [*t*(20.93) = −0.60, *p* = 0.558] between the dominant and non-dominant carrier language groups.

Before examining individual differences, we conducted a sensitivity power analysis using the *pwr* package (Champely, 2020). With our sample size, we had 80% power to detect correlations of *r* = 0.55 for pairs including learning-phase measures (*N* = 23), and *r* = 0.54 for the others. Thus, we had sufficient power to detect only large correlations. We examined Pearson correlations among child, maternal, and task measures (see Table S1). The significant associations all involved maternal factors and children’s performance in the single-language condition, and we followed up these three associations with partial correlations controlling for related background variables (see Table S2). Controlling for child age, receptive vocabulary in both languages, and the remaining maternal factor (mother’s education or language mixing), neither maternal factor was significantly associated with children’s attention during learning in the single-language condition (mother’s education: partial *r* = −0.42, *p* = 0.070; mother’s language mixing: partial *r* = −0.41, *p* = 0.082). However, mother’s language mixing was negatively associated with children’s target looking during the test in the single-language condition, and this association remained significant in the fully adjusted model (partial *r* = −0.51, *p* = 0.021).

## 4. Discussion

The present study examined the effect of language mixing on novel word learning in 3- to 4-year-old Korean–English bilingual children, an untested population simultaneously learning two distant languages. Overall, children could learn the novel word when it was presented in a single-language context but showed difficulty doing so under the mixed-language condition. In particular, the learning pattern emerged asymmetrically: language mixing was influential when learning and switching occurred in a non-dominant language context, whereas no such difference was observed when learning occurred in the dominant language context, though this asymmetry is preliminary given the small sample size. Neither children’s vocabulary scores in two languages nor differences in the mothers’ use of mixed language appeared to modulate word learning under our task conditions. Furthermore, the difference in learning outcomes did not appear to stem from attentional focus during the learning phase: looking times did not differ between the single- and mixed-language conditions, or across levels of language dominance.

### 4.1. The Effect of Language Mixing on Novel Word Learning

The negative effect of language mixing observed here partially replicates and extends the findings of Byers-Heinlein et al. (2022), who reported similar costs for French–English bilingual 3-year-olds, to a same-age Korean–English bilingual population. Also, our finding appears overall consistent with a body of research documenting the cognitive costs that language mixing imposes on bilingual children’s language processing and word learning (Byers-Heinlein et al., 2017; Gross et al., 2019; Morini & Newman, 2019; Niu et al., 2024; Potter et al., 2019). French–English bilingual 20-month-olds recognized the familiar referent better in a single-language context than in an intra-sentential code-switched context (Byers-Heinlein et al., 2017). Spanish–English bilingual 18-to 24-month-olds were also better able to recognize familiar words in a single-language sentence frame than in a code-switched frame (Morini & Newman, 2019). Potter et al. (2019) further demonstrated that intra-sentential code-switching incurred a cost in 18–30-month-old Spanish–English bilingual children’s familiar word recognition when switching occurred from dominant into non-dominant language. Similarly, 6–11-year-old Spanish–English bilingual children were slower in processing code-switched sentences than single sentences in an auditory moving window sentence processing task (Gross et al., 2019; see also Gross et al., 2021, for a disadvantage under a noisy environment), although their offline comprehension accuracy (i.e., responding to yes/no questions) was not different. When learning new words, 4-year-old Chinese bilingual learners showed a similar disadvantage when word learning occurred in a code-switched context (Niu et al., 2024). This negative impact on word learning and processing has been implicated in slower vocabulary accumulation among some bilingual populations (Byers-Heinlein, 2013), especially when the mixed input is provided by the same speaker (Carbajal & Peperkamp, 2020).

However, the negative effect we observed may reflect the specific experimental paradigm and the particular type of intra-sentential code-switching we used, rather than a generalizable hindering factor—a possibility also raised in our model study (Byers-Heinlein et al., 2022). In their study, in ours, and in the paradigm adopted by Niu et al. (2024), the target novel word always appeared downstream after encountering the code-switched familiar word (e.g., “There is a [tokki] (familiar word) on the jonu (novel word)”) and was accompanied by both familiar and novel referents. Because this paradigm requires multiple referents to be presented simultaneously, their presence—in addition to the demands of processing a code-switched word—may have compounded the task’s difficulty, making it cognitively demanding to encode and retain the novel label, as other word learning studies have shown (e.g., Horst & Samuelson, 2008; Samuelson & McMurray, 2017).

### 4.2. Modulating the Cost of Language Mixing on Word Learning

Crucially, the negative influence of language mixing emerged specifically when children learned novel labels in their less dominant language. Children mapped novel labels to referents successfully in the single-language context but had difficulty when the target label followed a code-switched familiar word from their dominant language (e.g., [tokki] on the Jonu; [Bunny]-ga Jonu wi-e). That is, difficulty arose when children had to switch from their dominant language to their less dominant one to encode the novel label. A parallel asymmetry has been reported in familiar word recognition among toddlers, with higher costs for dominant-to-non-dominant switches (Byers-Heinlein et al., 2017; Potter et al., 2019), and in unbalanced bilingual adults’ sentence comprehension (Bultena et al., 2015a, 2015b).

One possible account of this asymmetric cost draws on the cognitive demands of switching into the non-dominant language. This elevated cost may have temporarily hindered encoding and retention of the novel label. Processing non-dominant language input is itself more cognitively demanding (Declerck & Koch, 2023; Gross et al., 2021), and the added presence of a language switch may have compounded this burden. When a language switch occurs mid-sentence, lexical items and structural rules from both languages are simultaneously co-activated, requiring ongoing management of cross-language competition. As Hofweber et al. (2019) and Treffers-Daller et al. (2020) have argued, intra-sentential language mixing of this kind places particularly high demands on conflict monitoring, as the bilingual must continuously manage co-activated and competing lexico-structural representations from two languages. When this conflicting load operates on top of the already increased demands of non-dominant language processing, the cognitive resources required for novel word encoding may be depleted, leaving insufficient resources to establish new word–referent mappings. Niu et al. (2024) similarly argued that bilingual children’s difficulty with learning novel words in mixed-language sentences reflects the cognitive demands of simultaneously activating and suppressing two language systems, leaving insufficient resources for encoding and retaining new word–referent mappings. Because the present study did not directly measure cognitive control or inhibition, however, this interpretation should be treated with caution, and future work is needed to assess these processes directly. Notably, this type of processing asymmetry was not observed with school-aged children (Gross et al., 2019), suggesting that the asymmetric processing cost may vary by age, task type, or other factors, such as language experience (e.g., Chan & Kroll, 2026).

Recent studies, in fact, have not found negative effects of language mixing on word learning in other types of learning situations (Kaushanskaya et al., 2023; Libersky et al., 2025; Tsui et al., 2023). Kaushanskaya et al. (2023) taught novel labels to 4- to 5-year-old Spanish–English bilingual children using a single referent display, presenting each label four times in code-switched or single-language sentences. After the first trial, learning was comparable across conditions—and, surprisingly, children reached the learning criterion faster when labels were embedded in code-switched sentences than in monolingual sentences. Reducing task demands, as Kaushanskaya et al. (2023) did by allowing a single target referent to be mapped to the label with multiple repetitions, likely supported word learning beyond whether the language was code-switched. This was further demonstrated by Libersky et al. (2025), who used the same teaching paradigm. Four- to five-year-olds in their study learned nouns and verbs in the code-switched condition equally well as in the single-language condition. Similarly, it appears that language mixing may no longer impose a burden on learning when language switching occurs across sentences rather than within sentences (Tsui et al., 2023).

In our study, language mixing appeared to be neither a hindrance nor a help when children learned words in their dominant language. This pattern resembles that of the Spanish–English bilinguals in Byers-Heinlein et al. (2022), who showed no clear evidence of word learning in either single- or mixed-language contexts. The divergence between their French–English and Spanish–English samples may in turn reflect other differences between the groups, such as socioeconomic background, as also reported in Kremin et al. (2023). In the current study, however, the two dominance-based subgroups did not differ in SES, vocabulary size, or maternal language mixing, suggesting that language dominance may shape learning in more subtle ways than these factors capture (cf. Bultena et al., 2015a, 2015b). Nevertheless, the absence of significant learning in either sentence type in this group may reflect insufficient statistical power to detect a learning effect, given the small subgroup sample size. These results should therefore be interpreted with caution, and larger samples and other bilingual populations, with these variables controlled, are needed to re-test this possibility and corroborate the independent role of language dominance.

There are other conditions under which code-switched sentences may not negatively affect word learning. Kremin et al. (2023), for instance, found that mid-sentence code-switching did not hinder novel noun learning when the switch occurred within functionally uninformative elements—such as determiner–adjectival modifiers (e.g., …le bon duck)—that were unnecessary for referent resolution (e.g., when only one duck was available). This suggests that the disruption caused by intra-sentential language mixing may be limited to semantically important content words such as nouns (see Fernández Fuertes et al., 2025, for a related report).

### 4.3. The Role of Typological Language Distance

The typological distance between a bilingual’s two languages may also matter. Niu et al. (2024) found that Chinese–English bilinguals mapped labels to referents more successfully than Chinese–Japanese bilinguals, suggesting that mixed-language input is less disruptive when the two languages are typologically distant. Drawing on a lexical measure of language distance based on phonological similarity, Niu et al. (2024) argued that the more closely related a bilingual’s two languages are, the greater the cognitive load that language mixing imposes on word learning—such that children acquiring typologically similar language pairs should be more disrupted by mixed-language input. Under this account, Korean–English bilingual children, whose languages are more typologically distant than the French–English and Spanish–English pairs tested in Byers-Heinlein et al. (2022), should show relatively less interference from language mixing and thus learn novel words more successfully from code-switched sentences. The present data do not fit this prediction: Korean–English bilingual children’s word learning was disrupted only by language switching from dominant to non-dominant language. This raises the possibility that the directionality of code-switching may matter more than typological language distance, given the specific word learning context used in our study. However, the switching cost observed in our study could be related to the language distance, with a possibility that both factors together shape variable learning outcomes in an interactive way.

It should be noted, however, that the role of language distance has been tested directly in only one study (Niu et al., 2024) thus far. Notably, the Chinese bilinguals in that study were short-term L2 learners: both groups had acquired their second language within the preceding year in a preschool setting, which limits generalization to simultaneous bilinguals. To address this gap, we tried to match our sample and experimental design as closely as possible to those of Byers-Heinlein et al. (2022), so that the role of language distance could be compared and examined. Some differences nevertheless constrain direct comparison with the French–English and Spanish–English samples, including the position of the target noun within the sentence (sentence-medial vs. sentence-final) and details of the laboratory setup. Our study also manipulated the language of the carrier phrase, presenting it in either the dominant or the non-dominant language across participants, whereas Niu et al. (2024) used Mandarin as the carrier phrase language throughout. Given these differences in language learning context, treatment of language dominance, and participant age, the divergence in findings is more plausibly attributed to population- and design-specific factors than to language distance per se. Systematic control of these variables in future work will be needed to clarify how language distance shapes bilingual children’s word learning from mixed-language input.

### 4.4. Limitations and Future Directions

Other limitations of the present study should also be noted. First, although the stimuli and procedures were largely adapted from prior work to facilitate direct comparison, the language structure inevitably constrained the location of the target word. In Byers-Heinlein et al. (2022), the novel target word occupied sentence-final position in both language versions; the verb-final structure of Korean made this impossible here, and we could only ensure that the novel word consistently followed the familiar word in both languages. As noted above, the novel word may therefore have been perceptually less salient in the Korean sentences than in the original stimuli—plausibly contributing to the less robust learning observed here relative to the French–English sample and further limiting direct comparison across studies. Future work adapting this paradigm to typologically distant language pairs should develop stimuli that allow the novel word to occupy a consistent and salient sentential position across both languages.

Second, like prior experimental studies of language mixing, the present study presented experimenter-designed mixed-language sentences in a controlled laboratory setting. Naturalistic contexts may either attenuate or amplify the effects observed here. On one hand, the switches that occur naturally in everyday speech appear to carry relatively little cost for children’s learning (Tsui et al., 2023) and are often deployed by parents in ways that scaffold comprehension and learning (Kremin et al., 2022; Ruan et al., 2023). By comparison, real-world simulated listening environments, by introducing noise conditions, revealed that bilingual children had greater difficulty processing mixed-language sentences (Gross et al., 2021). Laboratory paradigms may thus overestimate or underestimate the disruption children encounter in daily life, and future work should pursue more ecologically valid designs.

Third, our sample is limited in representativeness. Participants were recruited primarily from Seoul and its metropolitan area, and the mothers in our sample had relatively high levels of education (see Table 1). The present findings may therefore not generalize to the broader population of Korean–English bilingual children with respect to geographic region, parental education, and socioeconomic status. Further studies with geographically and socioeconomically diverse samples are needed.

Fourth, the present sample was modest in size. The between-group comparison central to our dominance-based findings therefore rests on small cells, which limits the precision and reliability of these estimates. The dominance-based asymmetry we report should accordingly be regarded as preliminary, and its robustness remains to be confirmed in future studies with larger samples.

Finally, the individual difference factors—namely, children’s vocabulary level and maternal language mixing—considered in our study were limited. Nonetheless, our exploratory partial correlation analyses showed that mothers’ language mixing was negatively associated with children’s word recognition in the single-language condition, and this remained after controlling for child age, vocabulary, and mothers’ education. This pattern should be interpreted with caution give our small sample, but its direction is consistent with a growing body of work on the individual and contextual factors that shape bilingual children’s performance. Blom and van Witteloostuijn (2026) have identified attention as a key individual difference variable that moderates bilingual children’s performance on language-related tasks, and Byers-Heinlein et al. (2025) and Castellana and Benitez (2026) have emphasized that bilingual development is shaped not only by the quantity and quality of language experience, but by a multilayered system of interacting family, educational, and sociocultural factors. Although the present study did assess mothers’ language mixing, it did not capture how much mixed input children are actually exposed to in daily life or what types of language mixing parents use specifically. Future work should examine a broader range of sociocultural context variables, including the frequency and types of code-switching at home and in the community, family language policies, and the sociolinguistic status of each language in the community, to more systematically assess the generalizability of the present findings.

## 5. Conclusions

The present study provides new exploratory evidence that extends the hindering effect of language mixing on word learning to Korean–English bilingual children, and that this effect is further modulated asymmetrically by the direction of code-switching. Our results suggest that the negative consequences of language mixing for word learning are not limited to specific language pairs but may generalize across a broader range of bilingual populations, while also pointing to language dominance as a theoretically meaningful moderator of language mixing effects.

Beyond their theoretical relevance, our results also carry practical implications, as growing enthusiasm for early English education among Korean parents has led an increasing number of caregivers to mix Korean and English within sentences in their conversations with their children (Han & Kim, 2022). Whether such input helps or hinders early word learning is a question our controlled findings can only begin to address, and one that warrants direct study in naturalistic settings.

Most importantly, the present study was the first attempt to investigate the effects of language mixing in word learning among Korean–English bilingual children. Despite the growing body of research on bilingual language development, experimental work with this population remains notably scarce. In this regard, the present findings lay the groundwork for future research on Korean–English bilingual children’s language development and other related bilingual populations.

## Figures and Tables

**Table 1:** Demographics of participants, split by the two carrier phrase language conditions.

	Carrier Phrase Language		
	Total	Dominant	Non-Dominant
*N*	24 (10 boys)	12 (4 boys)	12 (6 boys)
Age (months)	41.88 (4.24)	41.50 (4.15)	42.25 (4.48)
Receptive Vocabulary			
Korean ^1^	96.33 (8.55)	94.42 (4.70)	98.25 (11.08)
English ^2^	101 (9.79)	101.3 (7.44)	100.8 (12.05)
Mother’s Language Mixing Score ^3^	10.67 (6.07)	11.25 (6.03)	10.08 (6.32)
Mother’s Years of Education	17.29 (2.26)	17.25 (2.45)	17.33 (2.15)

Note. Standard deviations are in parentheses. ^1^ *Receptive and Expressive Vocabulary Test* (standard score). ^2^ *Peabody Picture Vocabulary Test, 4th edition* (standard score). ^3^ *The Language Mixing Questionnaire*, a scale from 0 (no language mixing) to 30 (frequent language mixing).

**Table 2:** Hierarchical linear mixed-effects models predicting target referent looking time (logit-transformed).

	Model 1			Model 2			Model 3		
	*b*	*SE*	*t*	*b*	*SE*	*t*	*b*	*SE*	*t*
Fixed effects									
Intercept	−0.34	3.11	−0.11	−7.17	6.10	−1.18	−3.22	6.29	−0.51
Attention during learning	1.44	4.41	0.33	2.00	4.26	0.47	0.16	4.20	0.04
Language mixing	−1.39	0.82	−1.69 ^†^	−1.39	0.82	−1.70 ^†^	−1.40	0.82	−1.71 ^†^
Dominance (carrier language)	1.52	1.05	1.45	1.54	1.02	1.52	1.38	0.98	1.42
Language mixing × dominance (carrier language)	4.65	1.65	2.83 **	4.66	1.64	2.84 **	4.66	1.63	2.86 **
Dominant receptive vocabulary	–	–	–	0.008	0.031	0.26	0.005	0.030	0.17
Non-dominant receptive vocabulary	–	–	–	0.057	0.038	1.52	0.051	0.037	1.39
Mother’s language mixing	–	–	–	–	–	–	−0.147	0.089	−1.66
Random effects									
*SD* (intercept|participant)	1.46			1.29			1.11		
*SD* (residual)	3.71			3.70			3.68		
*Model fit*									
AIC	477.3			479.1			478.4		
BIC	494.3			500.9			502.6		
*p* (LRT)	–			2.24 (2)			2.65 (1)		
Δ*χ*^2^(df) Δ*χ*^2^(df) Δ*χ*^2^(df)	–			0.327			0.103		

Note. *N* = 83 observations and 23 participants (one participant was excluded due to missing data in the learning phase). All models estimated via maximum likelihood (ML) with a random intercept by participant. Degrees of freedom estimated via Satterthwaite’s approximation. Sentence type and dominance are effect-coded (±1). *b* = unstandardized coefficient; *SE* = standard error. The Sentence type × Dominance interaction remained significant across all three models. Model equations in R syntax: Model 1: *target looking time during test ~ language mixing × dominance (carrier language) + attention during learning + (1|id)*; Model 2: *dominant receptive vocabulary + non-dominant receptive vocabulary*; Model 3: *+ mother’s language mixing*. ^†^ *p* < 0.10. ** *p* < 0.01. Single-language condition [*M* = 0.69, *SD* = 0.29, *t*(11) = 2.39, *p* = 0.036]; tendency toward below-chance performance in the mixed-language condition [*M* = 0.33, *SD* = 0.29, *t*(10) = −1.92, *p* = 0.084].

## Data Availability

The data used in the current study are not available due to privacy restrictions. Requests to access the data should be sent to the corresponding author.

## Supplementary Materials

The following supporting information can be downloaded at https://www.mdpi.com/article/10.3390/bs16091547/s1: Table S1: Pearson correlations among child, mother, and task measures. Table S2. Partial correlations for the three associations that were significant at the uncorrected threshold.
