# Supplementary material for: Asymmetrical Effects of Language Mixing in Bilingual Children’s Word Learning
Source: Behav Sci (Basel). 2026 Sep 1;16(9):1547. doi: 10.3390/bs16091547 (PMC13603895; doi:10.3390/bs16091547)
Supplement: Supplementary file 1 [file behavsci-16-01547-s001.zip › behavsci-4488808-supplementary.pdf]

Supplementary Materials

Table S1. Pearson correlations among child, mother, and task measures.

Measure	1	2	3	4	5	6	7	8
1. Age (months)								
2. Receptive vocabulary (dominant language)	−.04							
3. Receptive vocabulary (non-dominant language)	−.03	−.06						
4. Mother's language mixing	.14	−.07	−.13					
5. Mother's education	−.16	.17	−.07	.30				
6. Attention during learning (single-language)	−.11	.03	.03	−.51*	−.48*			
7. Attention during learning (mixed-language)	−.17	.21	−.03	.01	.08	.38		
8. Target looking at test (single-language)	−.16	.01	.23	−.52**	−.04	.27	−.01	
9. Target looking at test (mixed-language)	−.18	.08	.11	.12	.06	.11	−.11	.24

Note. Receptive vocabulary was assessed with the PPVT-4 and REVT, assigned to the dominant and non-dominant language according to each child's dominance classification. Mother's language mixing was measured with the Language Mixing Questionnaire (Byers-Heinlein, 2013). Attention during learning refers to the mean proportion of looking to the screen during learning trials in each condition; target looking at test refers to the mean proportion of looking to the target referent during test trials in each condition. * $p < .05$; ** $p < .01$.

Table S2. Partial correlations for the three associations that were significant at the uncorrected threshold.

Association	Zero-order *r* (*p*)	Adjusted partial *r* (*p*)
Mother's language mixing × Attention during learning (single-language)	−.51 (.013)	−.41 (.082)
Mother's language mixing × Target looking at test (single-language)	−.52 (.009)	−.51 (.021)
Mother's education × Attention during learning (single-language)	−.48 (.021)	−.42 (.070)

Note. Partial correlations control for child age, receptive vocabulary in the dominant and non-dominant languages, and the maternal factor (mother's education for the language-mixing associations; mother's language mixing for the education association; four covariates in total, *df* = 17-18).
